# Experimental Investigation of the Piezoresistive Properties of Cement Composites with Hybrid Carbon Fibers and Nanotubes

**DOI:** 10.3390/s17112516

**Published:** 2017-11-02

**Authors:** Seung-Jung Lee, Ilhwan You, Goangseup Zi, Doo-Yeol Yoo

**Affiliations:** 1 Future Strategy Center, Korea Railroad Research Institute, 176 Cheoldobangmulgwan-ro, Uiwang-si, Gyeonggi-do 16105, Korea; seungjunglee@krri.re.kr; 2 School of Civil, Environmental and Architectural Engineering, Korea University, 145 Anam-ro, Seongbuk-gu, Seoul 02841, Korea; ih-you@korea.ac.kr (I.Y.); g-zi@korea.ac.kr (G.Z.); 3 Department of Architectural Engineering, Hanyang University, 222 Wangsimni-ro, Seongdong-gu, Seoul 04763, Korea

**Keywords:** cement-based sensor, carbon fibers, multi-walled carbon nanotubes, hybrid fillers, electrical resistivity, fractional change of resistivity, gauge factor, percolation threshold

## Abstract

Cement-based sensors with hybrid conductive fillers using both carbon fibers (CFs) and multi-walled carbon nanotubes (MWCNTs) were experimentally investigated in this study. The self-sensing capacities of cement-based composites with only CFs or MWCNTs were found based on preliminary tests. The results showed that the percolation thresholds of CFs and MWCNTs were 0.5–1.0 vol.% and 1.0 vol.%, respectively. Based on these results, the feasibility of self-sensing composites with four different amounts of CFs and MWCNTs was considered under cyclic compression loads. When the amount of incorporated CFs increased and the amount of incorporated MWCNTs decreased, the self-sensing capacity of the composites was reduced. It was concluded that cement-based composites containing both 0.1 vol.% CFs and 0.5 vol.% MWCNTs could be an alternative to cement-based composites with 1.0 vol.% MWCNTs in order to achieve equivalent self-sensing performance at half the price. The gauge factor (GF) for that composite was 160.3 with an R-square of 0.9274 in loading stages I and II, which was similar to the GF of 166.6 for the composite with 1.0 vol.% MWCNTs.

## 1. Introduction

As infrastructure deterioration increases, the demand for structural health monitoring (SHM) and prognoses that can evaluate and predict the service life of structures is growing rapidly [1,2,3,4,5,6,7]. Concrete infrastructure requires more maintenance than steel structures because of its non-homogeneous material characteristics. Numerous methodologies for SHM of concrete structures have been studied, such as strain/acceleration gauges, piezoelectric ceramic, fiber optic sensors, and shape memory alloys [1,2,3,4]. However, because most sensors are not made of concrete, they have poor compatibility with concrete. Metal sensors attached to or embedded in concrete can easily separate and corrode over time. The high cost of adhesive bonding and their low sensitivity and low survival rate are also drawbacks of such sensors [8,9].

A cement-based sensor that uses piezoresistivity to sense strain could be a good alternative to those sensors. Because concrete is considered an insulator, conductive fillers are incorporated to obtain conductivity for sensing [8,9,10,11,12,13]. Chen and Chung had been investigated carbon fiber reinforced concrete to monitor flaws in a concrete structure in 1993 [14]. Since 1993, numerous studies have been done on the composition and fabrication of such concrete sensors and measurement of their sensing signal, sensing properties, sensing mechanism, and applications [13,15,16,17,18,19,20]. In particular, traffic detection in pavement [15], damage detection and assessment of concrete structures [16], detection of dynamic strain behavior [17], and crack monitoring [18] have been tried using cement-based sensors with various nanomaterials. A cement-based sensor for ultra-high strength concrete [19] and sensitivity improvement techniques [20] have been investigated.

A cement-based sensor is clearly a complex structure because it is made by mixing many different materials. A cement-based sensor contains three microscopic phases: (1) interfaces between fillers; (2) cementitious matrix; (3) fillers. Among them, interfaces between fillers, which could form a network of conductive fillers, have the greatest effect on the electrical conductivity of a cement-based sensor [13,21]. Therefore, micro- or nano-scale fillers are commonly used, rather than macro-scale fillers, due to their enormous potential area for connecting with one another. More than 10 types of conductive fillers are available at the micro- or nano-scale, including carbon fiber (CF), graphite powder (GP), and nickel powder at the micro-scale and carbon nanofiber, carbon nanotube (CNT), titanium dioxide, and iron(III) oxide at the nano-scale [8,9,10,11,12,13,14,15,22,23,24]. 

One way to improve the potential area for connecting fillers is to mix different scales of fillers, forming hybrid fillers. A cement-based sensor with hybrid fillers offers improved self-sensing capacity, such as sensing reliability and sensitivity, through the interblend and interfinger effect of two kinds of dissimilar fibers [25]. Also, because nano-scale conductive fillers are more expensive than those at the micro-scale, more than 10 times expensive as in Korea, a cement-based sensor using hybrid fillers has an economic advantage. 

Azhari and Banthia [26] had been investigated the cyclic compression loading behavior of a cement-based sensor with 15% CFs and 1% multi-walled CNTs (MWCNTs). They found that a cement-based sensor with hybrid fillers showed an improvement in repeatability and accuracy compared to cement-based sensor with 15% CFs. Luo et al. [25] had been reported a cement-based nano-composite filled with 0.5% CFs and 0.1% MWCNTs, which is a huge difference from the work of Azhari and Banthia [26] in terms of filler content. Their cement-based sensor with hybrid filler had more linear and consistent variation along with cyclic change of stress. A cement-based strain sensor containing both CFs and carbon black (CB) had been investigated by Ou and Han [27] to improve the reproducibility of piezoresistivity under compressive strain such as a sensitivity of 0.0138%/με, a resolution of 0.007 με, a linearity of 4.25%, a repeatability of 4.36%, and a hysteresis of 3.63%. A cement-based sensor with CFs and GP was studied by Fan et al. [28]. They found that 1% CFs, 20% or 30% GP particles, and 4% cementitious capillary crystalline waterproofing materials in a cement-based composite improved the mechanical properties and stability of conductivity. Luo et al. [29] studied a cement-based nano-composite filled with 0.1% MWCNTs and 0.1% nano-phase CB, but it offered almost no improvement in self-sensing repeatability or variation stability. Using 2% polyvinyl alcohol fiber and 0.1%, 0.2%, or 0.5% CB has also been tried to improve self-sensing sensitivity [30]. Luo et al. revealed that spherical particle fillers are inclined to form aggregate chains in composites, in contrast with well distributed fibers [29]. As just discussed, several research groups have considered the feasibility of cement-based composites with hybrid fillers. However, no one has investigated the percolation threshold and optimal content of hybrid fillers.

Hybrid fillers should be on different scales and easy to obtain. CFs, which are widely used in other industries and therefore easy to obtain, could be a proper micro-scale filler for cement-based composites with hybrid fillers. Fiber-shaped nano-scale fillers such as MWCNTs improve conductivity more effectively than particle-shaped nano-scale fillers. Thus, this research experimentally investigated the feasibility of a cement-based sensor with both micro-scale CFs and nano-scale MWCNTs. The self-sensing capacity of cement-based composite with CFs or MWCNTs are first investigated to find the percolation threshold. Based on the experimental results for each filler, the feasibilities of self-sensing composites with four different hybrid filler contents were considered under cyclic compressive loads. The fractional changes in electrical resistivity were examined and compared according to cyclic compressive load and strain. The gauge factor was also investigated to compare the composites’ sensitivity.

## 2. Experimental Procedure

### 2.1. Preparation

Cement paste was adopted as the matrix for the cement-based sensors. Ordinary portland cement (OPC) and silica fume (SF) were used in a binary binder system, following previous works that used SF-enhanced dispersion to mechanically separate agglomerate MWCNTs [26,31]. The chemical and physical properties of OPC and SF are given in Table 1 [32,33].

**Figure 1:**
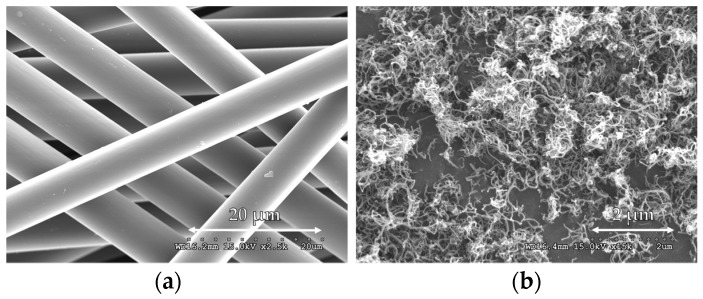
SEM images of CFs and MWCNTs; (**a**) CFs; and (**b**) MWCNTs.

The physical properties and costs of the CFs and MWCNTs considered here as electrical conductors are shown in Table 2 and Figure 1. The CFs and MWCNTs were produced by ACE C & Tech Co., Ltd. and Carbon Nano-material Technology Co., Ltd., respectively. MWCNTs are around five times more expensive than CFs at the same vol.%, as shown in Table 2. MWCNTs agglomerate together due to high van der Waals forces [34], as shown in Figure 1. A sonicator (QSONICA. Q500) was used to achieve good dispersion of the MWCNTs. The chosen amounts of MWCNTs and distilled water were put in a beaker, and the mixture was sonicated with a 10 s break every 1 h. Distilled water was used instead of tap water in order to prevent chemical reactions caused by impurities [31]. This solution was used as a solvent for the cement-based composite with hybrid fillers.

A set of specimens was prepared to investigate self-sensing capacities. The mixture proportions used in this study are shown in Table 3. The water/binder ratio of all specimens was 0.35, and SF was used for 30% of the cement weight. The total replacement ratios of CFs and MWCNTs were 0.5% and 1.0% volume fractions, respectively. To evaluate their effects on sensing capacities, CFs and MWCNTs were incorporated simultaneously with different replacement ratios of 0.2, 0.43, 1.0, and 2.33 of CF/MWCNT.

As the first step in fabricating cement-based sensors, the binder, OPC, and SF were dry mixed for 5 min using a 120-L Hobart type mixer. Then, the CF was gradually incorporated into the dry mixture and additionally mixed for an additional 5 min. After that, the dispersion solution made using the sonicator was added, and a controlled amount of super plasticizer (determined in previous research) was added to optimize flowability (150 ± 10 mm) [32,33]. The complete mixture was mixed for an additional 5 min. After mixing, the composites were cast into 50 × 50 × 50 mm^3^ cubic molds.

### 2.2. Measurement

**Figure 2:**
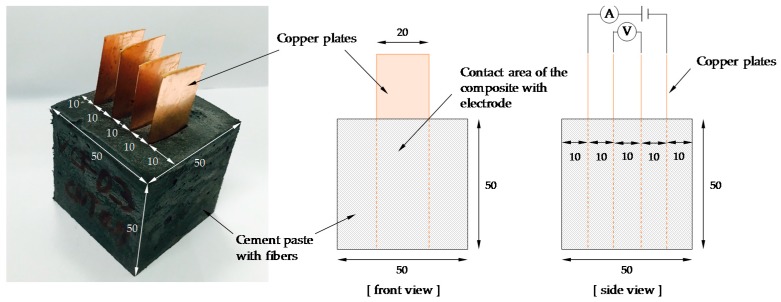
Test specimens for resistance measurement (unit: mm).

Since the four-probe method can eliminate contact resistance between electrodes, it is preferred to the two-probe method [13]. Thus, in this study, the four-probe method was used to measure electrical resistance, as shown in Figure 2. The 20 × 75 × 0.3 mm copper electrodes were inserted into the cement-based sensor with a spacing of 10 mm. The distance between two voltage poles was 10 mm. The contact area of the composite with an electrode was 1000 mm^3^. It needs to note that the compressive strength of cubes made of cement paste can be reduced by inserting copper plate, as reported by Han et al. [35]. Thus, the tested cubic specimens with four copper plates were only used for evaluating piezoresistive property not for compressive strength measurement. All specimens were cured at 23 ± 1 °C and 60 ± 5% relative humidity for 28 days. At days 7, 14, and 28, the electrical resistance was measured using a GWINTEK 819 LCR meter without an external load.

**Figure 3:**
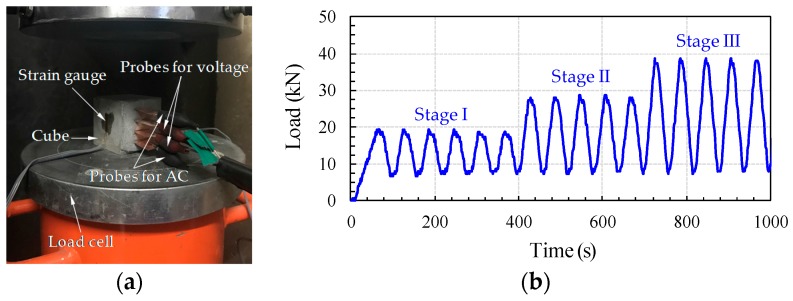
Cyclic compression test; (**a**) experimental set-up; and (**b**) loading protocol [32].

After 28 days, cyclic compression tests were carried out as shown in Figure 3 [32]. The cyclic compressive load was applied by an MTS 815 universal testing machine with the loading protocol shown in Figure 3b. The minimum compressive load was fixed to 10 kN to prevent the settlement effect. The change in resistance was measured and compared to the strain change, which was measured by a strain gauge.

**Figure 4:**
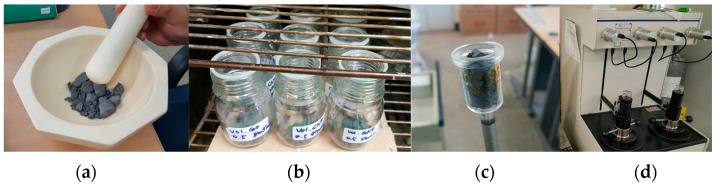
Mercury Intrusion Porosimetry (MIP) test; (**a**) fragments of crushed specimens; (**b**) fragments immersed in acetone; (**c**) fragments after drying; and (**d**) injection Mercury to measure pore volume.

The mercury intrusion porosimetry (MIP) test was carried out to investigate the porosity and pore size distribution of the cement-based composites. Figure 4 shows the detailed process for MIP testing. After the compressive test, 2–3 mm fragments were collected from the crushed specimens as shown in Figure 4a. To stop the hydration of the cement composite, the collected fragments were immersed in acetone for 24 h as shown in Figure 4b. Then, the fragments were dried in a thermo-hygrostat at 60 °C for 24 h. The fragments after drying is shown in Figure 4c. In the MIP test, pores are considered to be circular, so the pore diameter can be calculated using the well-known Washburn equation, *d* = −4γcos *θ*/*P*, where *d* is the pore diameter, *γ* is the surface tension (485 dynes/cm), *θ* is the contact angle (130°), and *P* is the injection pressure (0.1–33,000 psi). The volume of mercury that intrudes into the fragment could be measured at each injection pressure as shown in Figure 4d, and consequently, the pore diameter was calculated.

## 3. Self-Sensing Capacity of Cement-Based Composites with CFs

**Figure 5:**
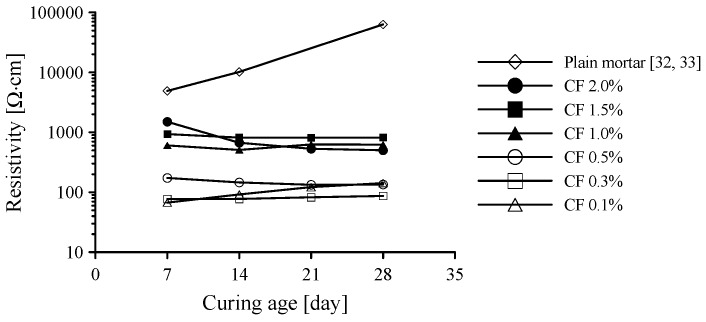
Effect of amounts of CFs for electrical resistivity with ages.

Electrical resistivity can be a simple indicator to evaluate the electrical performance of cement-based composites and can be calculated as follows:

*ρ = RA/l* (1)

where *ρ*, *R*, *A*, and *l* are electrical resistivity, resistance measured by LCR meter, contact area of the composite with the electrode, and distance between two voltage poles, respectively. The electrical resistivity of cement-based composites with six different CF contents is shown with curing age in Figure 5. The electrical resistivity of plain paste without any conductive fibers increased with curing age from 10,000 Ω·cm to 100,000 Ω·cm by 28 days because of the evaporation of pore water inside the cement paste, as reported previously [32]. This indicates that plain paste has no electrical conductivity. Because plain paste can thus be considered an insulator, conductive fillers are properly incorporated to obtain conductivity for self-sensing.

**Figure 6:**
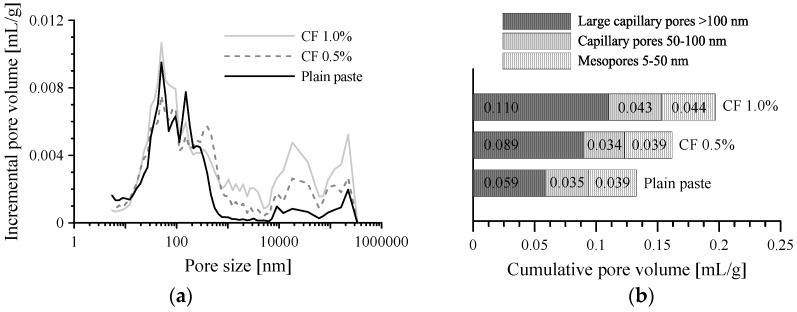
Porosity of cement-based composites with 0.5 and 1.0 vol.% CFs; (**a**) incremental pore volume; and (**b**) cumulative pore volume according to pore size.

**Figure 7:**
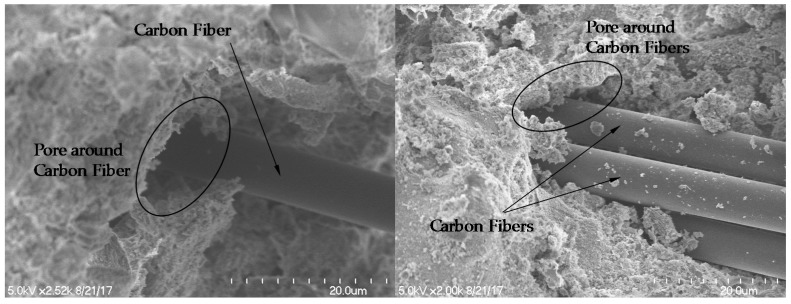
SEM images of cement-based composites with CFs.

When CFs were incorporated into the paste, the electrical resistivity was significantly reduced compared to that of plain paste. The composite with CFs had no significant change in resistivity with curing age, in contrast with plain paste, as shown in Figure 5. It is notable that the composite with 1.0–2.0 vol.% CFs had 10 times higher electrical resistivity than that with 0.1–0.5 vol.% CFs. In particular, as given in Figure 5, the resistivity of cement composites with CFs increased greatly as the volume fraction of CFs increased from 0.5% to 1%. This phenomenon shows that adding CFs improves the conductivity of cement paste for sensing, but more than 1.0% of CFs causes a detrimental effect to the conductivity of the paste, which might be caused by the increased porosity that results from an increasing amount of CFs. To investigate the effect of CFs content on electrical resistivity, the porosity was measured by the MIP test, as shown in Figure 6. The incremental pore volume for paste with 1.0 vol.% CFs was larger than that for paste with 0.5 vol.% CFs and plain paste, as shown in Figure 6a. In addition, the incremental pore size was summed and then divided into three representative sizes based on previous research, as shown in Figure 6b [21,36]: (1) mesopores (5–50 nm), which generally indicate hydration products such as C-S-H; (2) capillary pores (50–100 nm); (3) large capillary pores (>100 nm), which could affect the strength and permeability of cement paste through pores between cement particles or in the interfacial transition zone (ITZ). The presence of CFs enlarged the large capillary pores, though the volume of mesopores in the three specimens was almost the same. Therefore, the cement paste with 1.0 vol.% CFs had a total porosity of 0.197 mL/g, about 48.1% and 21.0% higher than that of plain paste (0.133 mL/g) and cement paste with 0.5 vol.% CFs (0.162 mL/g), respectively. Because the CFs were not well dispersed and stuck together in the cement paste in the well-known fiber balling phenomenon, large capillary pores formed between the CFs and the cement paste. This result is similar to the research of Li et al. [37]. They observed that cement paste with 0.5% CFs had a total porosity of 23.4%, about 31% higher than that of cement paste without fibers, whereas cement paste with 0.5% MWCNTs had a total porosity of 10.8%, about 64% lower than that of cement paste without fibers. The capillary pores (>50 nm) in cement paste with 0.5% CFs were 9.89%, about 2.7 times higher than that of cement paste without fibers. The SEM images of cement-based composites with CFs shown in Figure 7 support this explanation. The pores appear as dark spaces between the CFs and cement paste.

To investigate self-sensing repeatability, cyclic compression tests were carried out. The cyclic compressive load, which is an input of the test, and strain change measured by a strain gauge were compared to the fractional change of resistivity (FCR). If the contact area between the composite and the electrode and the distance between two voltage poles do not change during the test, FCR can be calculated as follows:

FCR = Δ*ρ*/*ρ*_0_ ≒ Δ*R*/*R*_0_ (2)

where Δ*ρ*, *ρ*_0_, Δ*R*, and *R*_0_ are change in electrical resistivity, initial electrical resistivity, change in resistance, and initial resistance measured by LCR meter, respectively.

**Figure 8:**
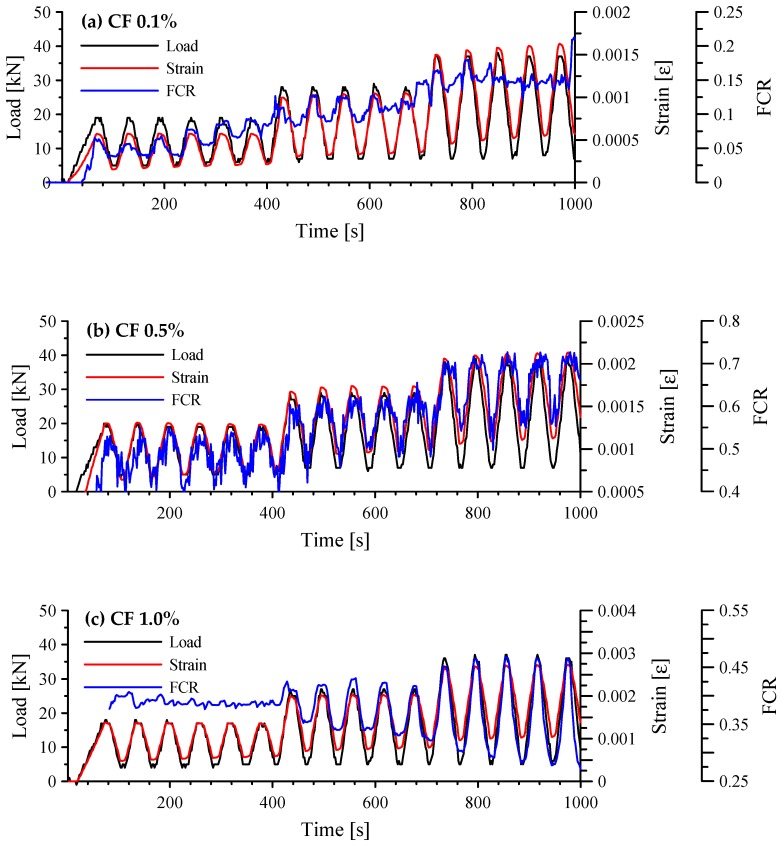
Responses of cement-based composites with 0.1, 0.5 and 1.0 vol.% CFs under cyclic compression; (**a**) composites with 0.1 vol.% CFs; (**b**) composites with 0.5 vol.% CFs; and (**c**) composites with 1.0 vol.% CFs.

The responses of cement-based composites with 0.1, 0.5, and 1.0 vol.% CFs under cyclic compression are shown in Figure 8. When an external compressive force is applied to the specimens, the change in resistance Δ*R* should be negative because resistance is reduced as the carbon fibers get closer to one another, which leads to the formation of more conductive pathways. The conductive pathways that allow electrical current to flow can be achieved by directly connecting the CFs and by the tunneling effect [32]. Thus, FCR multiplied by −1 was drawn in this figure to enhance readability. Also, strain in this figure indicates compressive strain. When 0.1 vol.% CFs were incorporated into the paste, FCR could not follow a cyclic trend in loading stages II and III, as shown in Figure 8a. Also, significant unintended noise occurred during the tests. Although the pore volume was probably smaller than in the composites with more than 0.5 vol.% CFs, the composite with only 0.1 vol.% CFs did not have enough electrical conductivity. The composites with 0.5 and 1.0 vol.% CFs followed a cyclic trend, as shown in Figure 8b,c, respectively. However, the FCR of the composite with 0.5 vol.% CFs also produced obvious unintended noise in every loading cycle, whereas that of the composite with 1.0 vol.% CFs had only minor fluctuations, especially at stage III. Increasing the amount of CF in the cement paste thus decreased noise during the tests, except during stage I in Figure 8c, which had unstable data come from the measurement error. It is interesting to note that the response tendency of the cement-based composites with 0.5 and 1.0 vol.% CFs under cyclic compression shown in Figure 8b,c does not correspond with that for electrical resistivity, shown in Figure 5. Although the cement paste with 1.0 vol.% CFs had 10 times larger electrical resistivity and 21.0% larger porosity than that with 0.5 vol.% CFs, any quantity larger than 0.5 vol.% CFs could be sufficient to produce sensing capacity. Therefore, when only CFs were incorporated in cement paste, the percolation threshold was between 0.5% and 1.0%.

The gauge factor (GF) was calculated as follows to evaluate the sensitivity of the strain sensor:

GF = (Δ*ρ*/*ρ*_0_)/*ε* (3)

where *ε* is compressive strain measured by a strain gauge, and Δ*ρ*/*ρ*_0_ is equal to the FCR calculated using Equation (2). 

**Figure 9:**
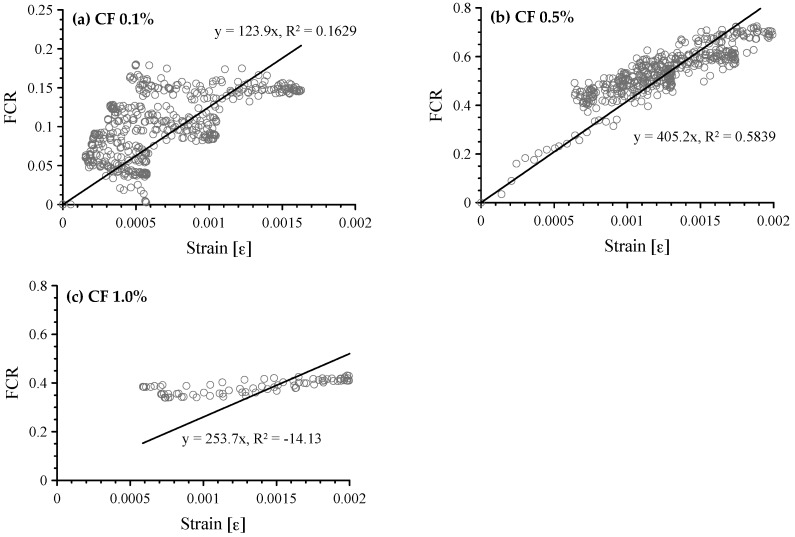
Correlation between FCR and compressive strain of cement-based composites with 0.1, 0.5 and 1.0 vol.% CFs; (**a**) composites with 0.1 vol.% CFs; (**b**) composites with 0.5 vol.% CFs; and (**c**) composites with 1.0 vol.% CFs.

The FCR vs. compressive strain of cement-based composites with 0.1, 0.5, and 1.0 vol.% CFs is shown in Figure 9. Linear regressions using the least square method are also shown in this figure. GF can be the slope of the regression line. As expected, when 0.1 vol.% CFs was incorporated in the paste, the FCR showed a scattering pattern and did not have a linear relationship with strain. However, the relationship between FCR and strain in the composites with 0.5 vol.% CFs was nearly linear with lower variability. The GF of 0.5 vol.% CFs was 405.2 with an R-square of 0.5839. Since the unstable data measured at stage I in the cement paste with 1.0 vol.% CFs, data from stage II and III are only plotted in Figure 9c. Since it showed a poor relationship and the chosen linear model with a zero y-intercept does not follow the trend of the data, the R-square was negative, −14.13.

## 4. Self-Sensing Capacity of Cement-Based Composites with MWCNTs

**Figure 10:**
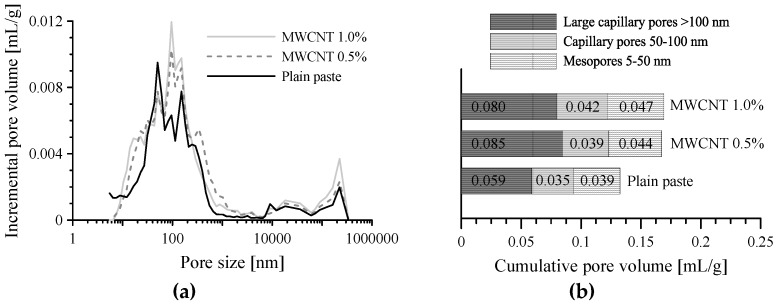
Porosity of cement-based composites with 0.5 and 1.0 vol.% MWCNTs; (**a**) incremental pore volume; and (**b**) cumulative pore volume according to pore size.

MWCNTs are nano-scale, unlike CFs, and they can be fixed tightly in cement paste. Increasing the amount of MWCNTs incorporated produced no significant increase in large capillary pores, unlike the cement paste with CFs, as shown in Figure 10. Thus, MWCNTs can act as a single conductive material with cement paste under load.

**Figure 11:**
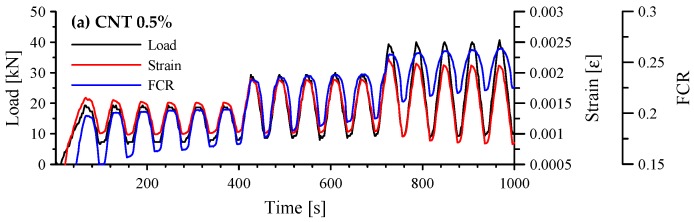

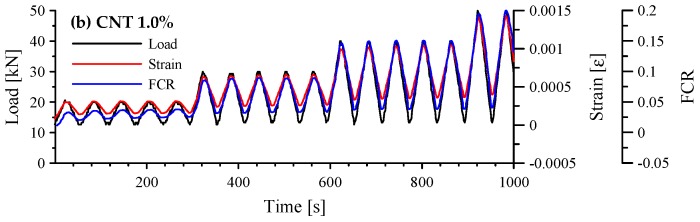
Responses of cement-based composites with 0.5 and 1.0 vol.% MWCNTs under cyclic compression; (**a**) composites with 0.5 vol.% MWCNTs; and (**b**) composites with 1.0 vol.% MWCNTs.

The responses of cement-based composites with 0.5 and 1.0 vol.% MWCNTs under cyclic compression are shown in Figure 11. When 0.5 vol.% MWCNTs were incorporated in the paste, the FCR fluctuated for 5–6 loading cycles and then stabilized in loading stage II, as shown in Figure 11a, because the compressive loads connected the fibers enough to produce conductivity. However, the variation in FCR decreased when the maximum compressive load increased in loading stage III. On the other hand, when 1.0 vol.% MWCNTs were incorporated in the paste, the FCR followed a cyclic trend in loading stages II and III, as shown in Figure 11b. Also, there was no residual FCR during the tests, which means that the paste with 1.0 vol.% MWCNTs had good self-sensing repeatability. That result has already been reported [32]. No unintended noise occurred during the testing of both specimens.

**Figure 12:**
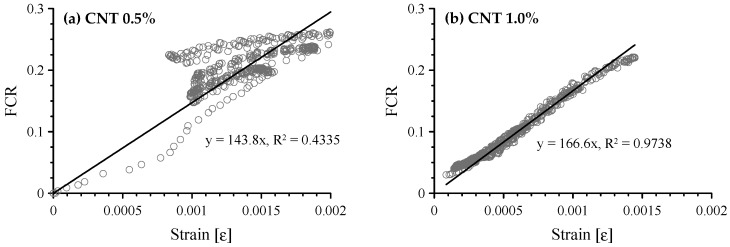
Correlation between FCR and compressive strain of cement-based composites with 0.5 and 1.0 vol.% MWCNTs; (**a**) composites with 0.5 vol.% MWCNTs; and (**b**) composites with 1.0 vol.% MWCNTs.

The FCR vs. compressive strain of cement-based composites with 0.5 and 1.0 vol.% MWCNTs is shown in Figure 12. When 0.5 vol.% MWCNTs were incorporated in the paste, the FCR showed a scattering pattern over a compressive strain of 0.001. Thus, GF, which is the slope of linear regression, was 143.8 with an R-square of 0.4335. However, the FCR had an almost linear relationship with strain for the composite with 1.0 vol.% MWCNTs, as expected. The GF was 166.6 with an R-square of 0.9738. 

Based on those results, the increase in MWCNTs in the cement paste increased composite sensitivity during cyclic compression tests. The paste with 1.0 vol.% MWCNTs is an adequate strain sensor. The only significant issue is the high cost of MWCNTs, as mentioned in the Introduction. Therefore, it would be better to use to hybrid fillers: decreasing the amount of MWCNTs needed by increasing amount of CFs to achieve equivalent sensing performance would solve the price problem.

## 5. Self-Sensing Capacity of Cement-Based Composites with Both CFs and MWCNTs

**Figure 13:**
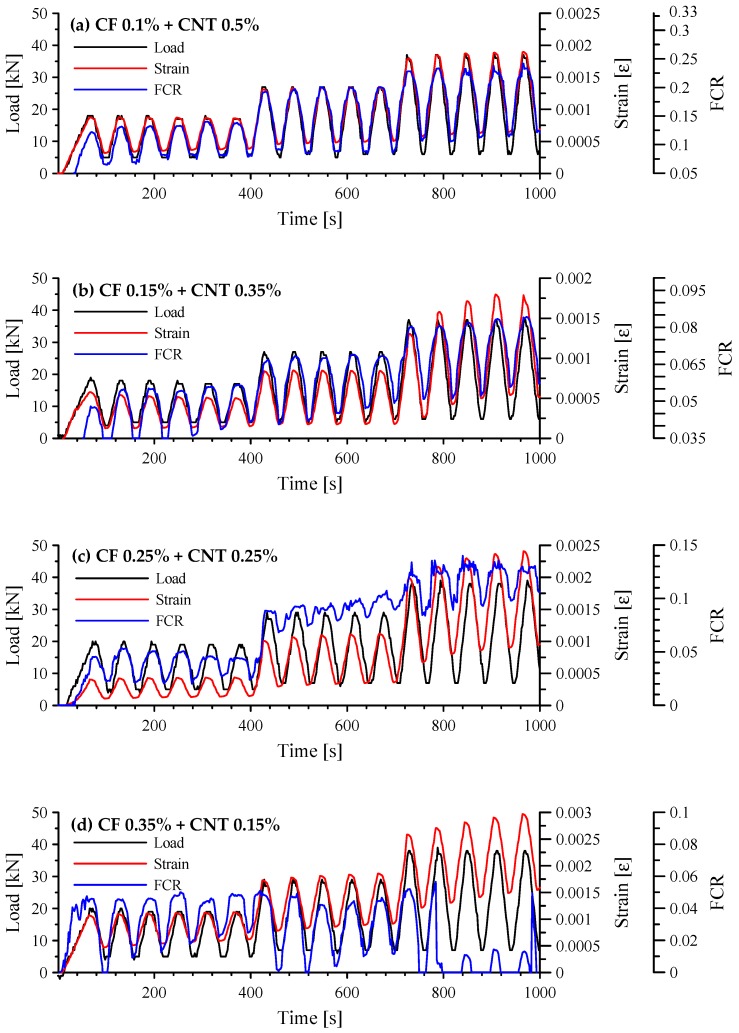
Responses of cement-based composites with both CFs and MWCNTs under cyclic compression; (**a**) composites with 0.1 vol.% CFs and 0.5 vol.% MWCNTs; (**b**) composites with 0.15 vol.% CFs and 0.35 vol.% MWCNTs; (**c**) composites with 0.25 vol.% CFs and 0.25 vol.% MWCNTs; and (**d**) composites with 0.35 vol.% CFs and 0.15 vol.% MWCNTs.

**Figure 14:**
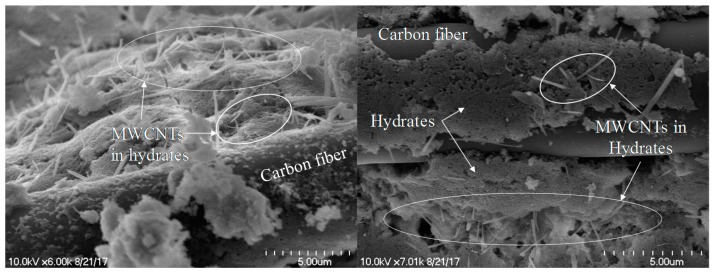
SEM images of cement-based composites with hybrid fillers as both CFs and MWCNTs.

The responses of cement-based composites with both CFs and MWCNTs under cyclic compression are shown in Figure 13. When 0.1 vol.% CFs and 0.5 vol.% MWCNTs were incorporated in the cement paste, the FCR followed a cyclic trend in all loading stages, as shown in Figure 13a. The response of this specimen was similar to that of the paste with 1.0 vol.% MWCNTs shown in Figure 11b. The cement-based sensor with hybrid fillers more effectively improved self-sensing capacity than that with one type of filler because of the interfinger effect of dissimilar fibers at different scales. The SEM images shown in Figure 14 support this explanation. The nano-scale MWCNTs connected to the micro-scale CFs, which offered enormous connection areas. Also, because there was no residual FCR during the tests, the paste with 0.1 vol.% CFs and 0.5 vol.% MWCNTs had self-sensing repeatability. When 0.15 vol.% CFs and 0.35 vol.% MWCNTs were incorporated in the paste, the FCR followed a cyclic trend in loading stage II, as shown in Figure 13b. Residual FCR appeared at the end of stage II and increased during the loading cycles. On the other hand, when the amount of incorporated CFs increased and the amount of incorporated MWCNTs decreased, the specimens had inadequate self-sensing capacity, as shown in Figure 13c,d. Unintended noise occurred at every loading stage. Thus, at least 0.35 vol.% of MWCNTs should be incorporated with CFs in order to produce sufficient conductivity. Also, because CFs can create large pores around the fibers, the amount of incorporated CFs should be as small as possible.

Since carbon is hydrophobic, many studies have been done to improve the dispersion and bond properties between CFs and cement pastes or polymer-based matrices [38,39,40,41,42]. The pores around CFs shown in Figure 7 were associated with the hydrophobicity of carbon. Fu et al. [43] investigated the FCR of cement paste including 0.24 vol.% CFs under compressive loads of 5263 cycles. They found that the FCR peaks decreased with increasing loading cycles until the mid-stage of the total cycles. They attributed the decrease in FCR peaks to separation between the CFs and cement paste caused by damage at the ITZ. The damaged ITZ can then cause the CFs to touch; thus, the FCR peaks decrease gradually. In this study, irreversible FCR peaks were found with an increase in the amount of CFs incorporated, as shown in Figure 13. On the other hand, MWCNTs are nano-scale and can be fixed tightly in cement paste, as shown in Figure 14. Thus, there was no significant change in porosity with changes in the amount of incorporated MWCNTs.

**Figure 15:**
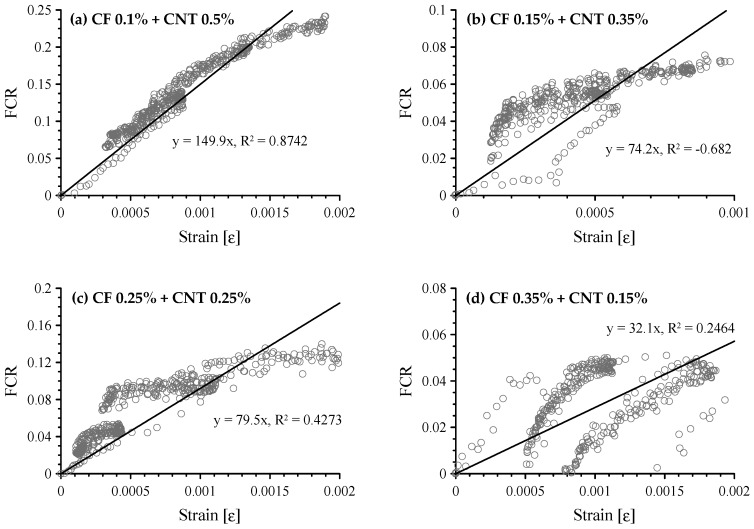
Correlation between FCR and compressive strain of cement-based composites with both CFs and MWCNTs; (**a**) composites with 0.1 vol.% CFs and 0.5 vol.% MWCNTs; (**b**) composites with 0.15 vol.% CFs and 0.35 vol.% MWCNTs; (**c**) composites with 0.25 vol.% CFs and 0.25 vol.% MWCNTs; and (**d**) composites with 0.35 vol.% CFs and 0.15 vol.% MWCNTs.

**Figure 16:**
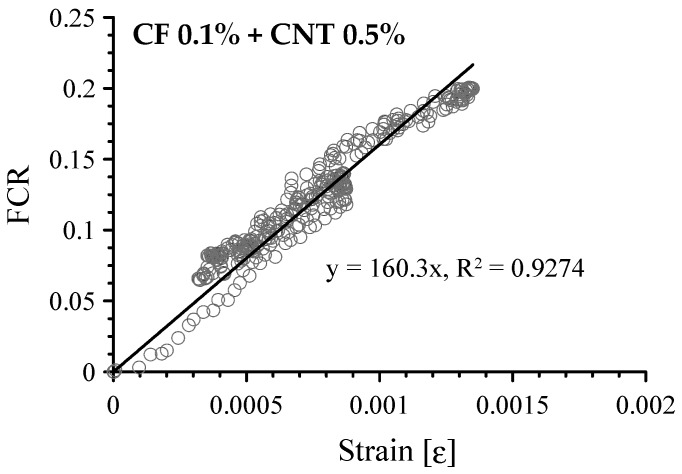
Correlation between FCR and compressive strain of cement-based composites with 0.1 vol.% CFs and 0.5 vol.% MWCNTs up to second stage of loading sequence.

The FCR vs. compressive strain of cement-based composites with both CFs and MWCNTs is shown in Figure 15. As expected, when 0.1 vol.% CFs and 0.5 vol.% MWCNTs were incorporated in the paste, the FCR had an almost linear relationship with strain, as shown in Figure 15a. Its GF was 149.9 with an R-square of 0.8742. In stages I and II, the GF was 160.3 with an R-square of 0.9274, shown in Figure 16, which is similar to the GF of 166.6 for the composite with 1.0 vol.% MWCNTs. It is also similar to GF (130) of cement-based sensor with 1.0 wt.% MWCNTs from the previous research by D’Alessandro et al. [22]. Figure 15b–d show scattered patterns and did not fit a linear regression. 

The GFs of cement-based composites with carbon materials examined in this study were much higher than that (about 2–5) of foil strain gauge. Thus, it can be noted that the cement-based sensors developed are more sensitive to strain under compressive force than the foil strain gauge commercially available. However, this does not imply that cement-based sensor is a better material for measuring the strain correctly than the strain gauge [44].

Both previous studies and these results indicate that MWCNTs are more effective than CFs in producing self-sensing properties. Although a large amount of CFs can lead to inferior sensitivity to piezoresistivity, a micro-conductive path can be secured with only a small amount of CFs. The deficiency in self-sensing sensitivity caused by the small amount of CFs can be compensated by fixing MWCNTs in the cement pastes. Therefore, a cement-based sensor with 0.1 vol.% CFs and 0.5 vol.% MWCNTs could be a proper solution for self-sensing that provides adequate sensing capacity at half the price of a cement-based sensor with 1.0 vol.% MWCNTs. Adding only 0.1 vol.% CFs to cement paste with 0.5 vol.% MWCNTs could improve the sensing capacity of cement paste with only 0.5 vol.% MWCNTs at a similar price. GFs obtained from the experiments are listed in Table 4. 

Reza et al. [45] reported that the electrical resistance of plain mortar and carbon fiber reinforced cement composites (CFRCC) is affected by the temperature and relative humidity. The resistance of both the plain mortar and CFRCC decreased with an increase in the temperature and its relationship was well fitted with the Hinrichson-Rasch law. On the other hand, the effect of relative humidity on the resistance was mitigated by including carbon fibers, meaning that there was no noticeable change of the resistance of CFRCC according to the relative humidity variation. Therefore, the effects of temperature and relative humidity on the electrical resistivity of cement-based sensors need to be thoroughly examined before their practical application real structures, and thus, a further study is required to be done.

## 6. Conclusions

This study experimentally investigated cement-based sensors with hybrid conductive fillers, both CFs and MWCNTs. The following conclusions are drawn from these results:

(1) Although a cement paste with 1.0 vol.% CFs had 10 times more electrical resistivity and 21.0% greater porosity than a paste with 0.5 vol.% CFs, the amount of incorporated CFs must be greater than 0.5 vol.% in order to produce an adequate piezoresistive sensing capacity. The percolation threshold of CFs is thus between 0.5 and 1.0 vol.% of the cement paste.
(2) Increasing the amount of MWCNTs in the paste increased the sensing sensitivity during cyclic compression tests. A 1.0 vol.% MWCNTs in the paste is sufficient for a strain sensor.
(3) The amount of MWCNTs incorporated with CFs should be at least 0.35 vol.% in order to produce sufficient piezoresistive sensing capacity. Also, the amount of incorporated CFs should be minimized to enhance sensing capacity.
(4) The cement-based sensor with 0.1 vol.% CFs and 0.5 vol.% MWCNTs had equivalent sensing performance to the composite with 1.0 vol.% MWCNTs at half the price. The GF is 160.3 with an R-square of 0.9274, which is similar to the GF of 166.6 for the composite with 1.0 vol.% MWCNTs.

## Figures and Tables

**Table 1:** Chemical compositions and physical properties of cementitious materials [32,33].

Composition % (Mass)	Cement	Silica Fume
CaO	61.33	0.38
Al_2_O_3_	6.40	0.25
SiO_2_	21.01	96.00
Fe_2_O_3_	3.12	0.12
MgO	3.02	0.10
SO_3_	2.30	-
Specific surface area (cm^2^/g)	3,413	200,000
Density (g/cm^3^)	3.15	2.10
Ig. loss (%)	1.40	1.50

Note: Cement = Type 1 Portland cement and SF = silica fume.

**Table 2:** Physical properties and cost of CF and MWCNT.

	Diameter (nm)	Length (mm)	Carbon Content (%)	Aspect Ratio	Density (g/cm^3^)	Cost * ($/vol.% of m^3^)
CF	10,000	6	93	>600	1.80	318
MWCNT	15	0.01	>90	>500	1.20	1695

* CF: 360,000 KRW/vol.% of m^3^ (http://www.aceca.co.kr/eng/index.php); MWCNT: 1,920,000 KRW/vol.% of m^3^ (http://www.carbonnano.co.kr/english/english.htm).

**Table 3:** Mix proportions used in this study.

Group		W/B	SF/OPC	CFs * [%]	MWCNTs * [%]	CFs/MWCNTs	SP ** [%]	Cost *** ($/m^3^)
Reference	Plain paste	0.35	0.3	-	-	-	-	-
CFs	CF0.1			0.1	-	-	-	31.8
	CF0.5			0.5	-	-	1.5	159
	CF1.0			1.0	-	-	1.9	318
MWCNTs	MWCNT0.5			-	0.5	-	1.0	847.5
	MWCNT1.0			-	1.0	-	1.6	1695
Hybrid	CF0.1CNT0.5			0.1	0.5	0.2	1.1	879.3
	CF0.15CNT0.35			0.15	0.35	0.43	1.3	640.95
	CF0.25CNT0.25			0.25	0.25	1.0	1.4	503.25
	CF0.35CNT0.15			0.35	0.15	2.33	1.4	365.55

Note: W/B = water-to-binder ratio; SF = silica fume; OPC = ordinary Portland cement; and SP = superplasticizer * Volume fraction ** Percentage of SP to binder, by weight *** Cost of fillers.

**Table 4:** Gauge Factor (GF) obtained from the experiments.

Group		GF
Reference	Plain Paste	
CFs	CF0.1	123.9 (R^2^ = 0.1629)
	CF0.5	405.2 (R^2^ = 0.5839)
	CF1.0	253.7 (R^2^ = −14.13)
MWCNTs	MWCNT0.5	143.8 (R^2^ = 0.4335)
	MWCNT1.0	166.6 (R^2^ = 0.9738)
Hybrid	CF0.1CNT0.5	160.3 (R^2^ = 0.9274) *
	CF0.15CNT0.35	74.2 (R^2^ = −0.682)
	CF0.25CNT0.25	79.5 (R^2^ = 0.4273)
	CF0.35CNT0.15	32.1 (R^2^ = 0.2464)
